# Tertiary syphilis and cardiovascular disease: the united triad: case report

**DOI:** 10.1093/ehjcr/ytae013

**Published:** 2024-01-06

**Authors:** Taha Berhil, Fatima Zohra Radi, Badre El Boussaadani, Zainab Raissouni

**Affiliations:** Cardiology Department, CHU Mohammed VI, Route de Rabat Km 17 BP 398, Gzinaya, Tangier, Morocco; Medical Cardiology Office of Dr Radi, Tangier, Morocco; Cardiology Department, CHU Mohammed VI, Route de Rabat Km 17 BP 398, Gzinaya, Tangier, Morocco; Cardiology Department, University of Abdelmalek Saadi, Tangier, Morocco; Cardiology Department, CHU Mohammed VI, Route de Rabat Km 17 BP 398, Gzinaya, Tangier, Morocco; Cardiology Department, University of Abdelmalek Saadi, Tangier, Morocco

**Keywords:** Tertiary syphilis, Ostial coronary, Aortitis, Case report

## Abstract

**Background:**

Syphilis, owing to its natural course, can lead to long-term damage to the aortic valve, such as insufficiency and rarely stenosis, ostial coronary stenosis, and syphilitic aortitis. Cardiovascular involvement alongside neurological involvement dominates the prognosis. This should no longer be seen, thanks to awareness and prevention programmes, medical treatment, and antibiotics.

**Case summary:**

We report a case of a 54-year-old chronic smoker with no previous history, admitted for respiratory distress amid an impaired general condition. An electrocardiogram was performed, which showed sinus rhythm with lateral ST depression and *T*-wave inversion. Coronary angiography revealed an ostial stenosis of the left coronary artery. Echocardiography displayed a globular dilated left ventricle with a left ventricular ejection fraction of 40% and severe aortic insufficiency (AI). Computed tomography angiography of the aorta showed a dilation of the thoracic aorta and suprarenal abdominal aorta. Syphilitic serology was positive. The patient underwent angioplasty, resulting in a satisfactory outcome, and subsequently received optimal treatment. Following a consultation with a cardiovascular surgeon and vascular team, it was decided to proceed with mechanical aortic valve replacement and aorto-coronary double bypass surgery, but vascular surgery of the ascending aortic aneurysm was not possible at once.

**Discussion:**

Tertiary syphilis should always be considered when faced with isolated coronary ostial involvement, aortic aneurysm, and/or AI. What makes our case special is that the patient had almost all the cardiovascular complications of tertiary syphilis. Primary syphilis should always be prevented, diagnosed early, and treated appropriately with antibiotic therapy.

**Learning points**

- Early screening for syphilis in high-risk populations can aid in the early detection and treatment of the disease, preventing further complications such as cardiovascular involvement.
- In instances of isolated coronary ostial stenosis, aortic aneurysm, and/or aortic insufficiency (AI)—particularly when these conditions coexist—tertiary syphilis should be strongly considered as a potential underlying cause.
- Valve replacement surgery and coronary bypass may still be necessary in cases of AI and ostial stenosis of syphilitic origin.

## Introduction

The World Health Organization estimated that in 2010, there were 11 million new cases of syphilis per year worldwide.^1^ In 1950–60, cardiovascular disease caused by syphilis was observed in <1% of autopsies.^2^ The incidence of syphilis has steadily declined, likely due to the prevalent use of penicillin.^3^ Cardiovascular involvement, along with neurological involvement, significantly affects the prognosis: issues can include aortic insufficiency (AI), thoracic aortic aneurysm, and ostial coronary artery disease.^4^ The diagnosis is often suggested by specific clinical signs and by a particular epidemiological context; it is typically based on serological methods.

This article presents an exceptionally rare case of a patient experiencing three cardiovascular diseases associated with tertiary syphilis.

## Summary figure

**Figure:**
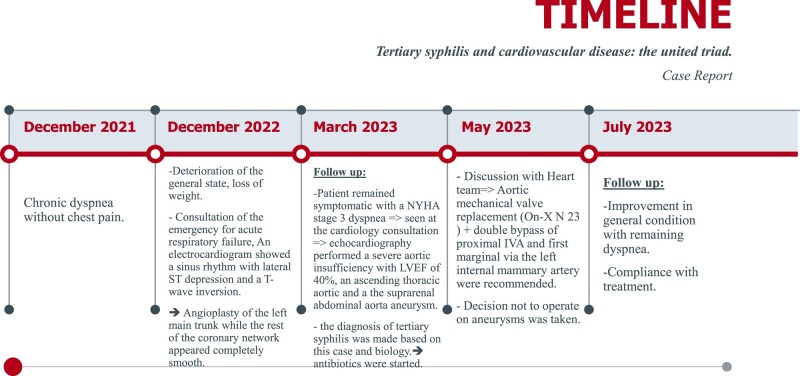


## Case description

**Figure 1:**
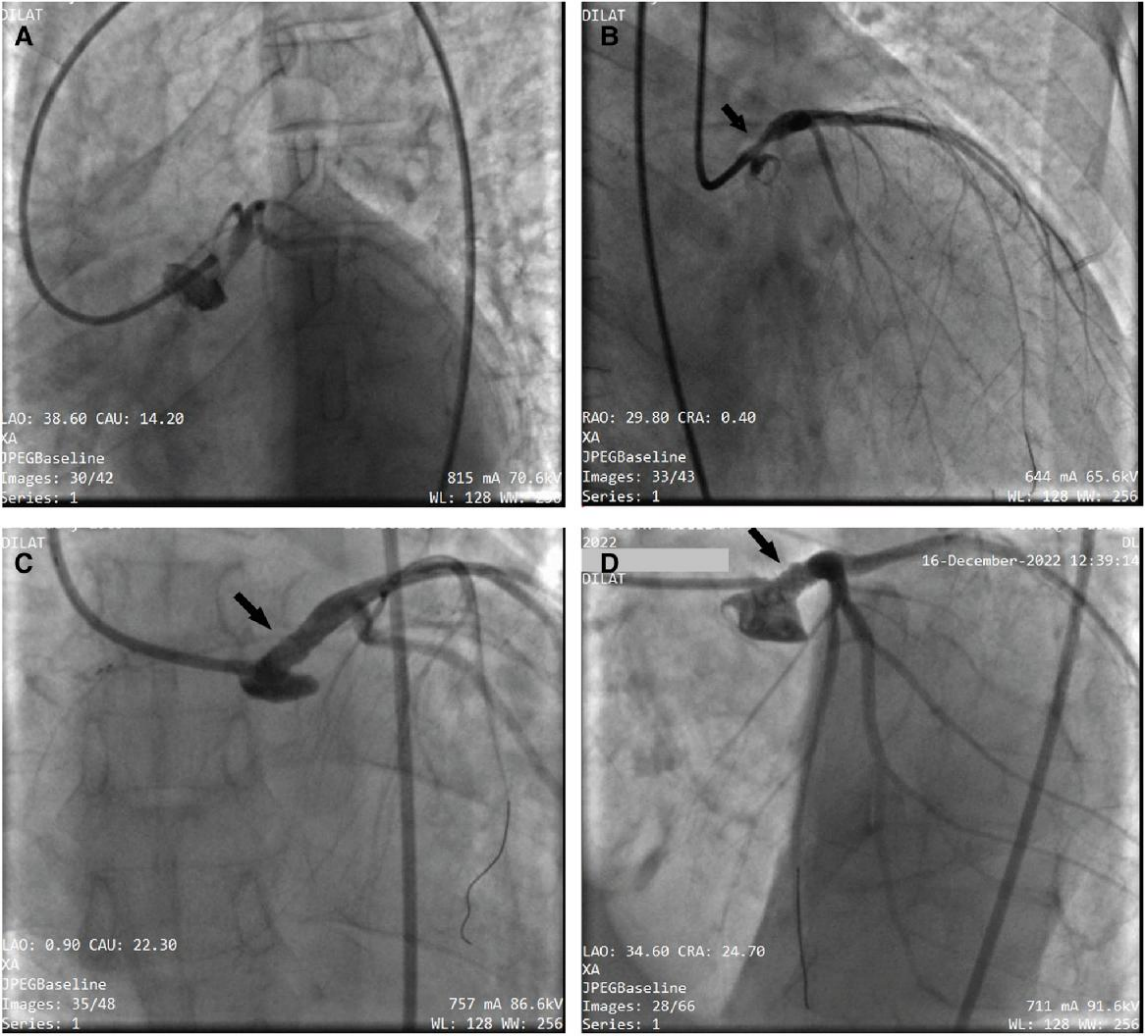
(*A* and *B*) Coronary angiography showing a very tight stenosis estimated at 80% of the ostial trunk (black arrow) with a smooth coronary network. (*C* and *D*) Result of coronary angioplasty of the common trunk with implantation of a 4 × 18 mm active stent (black arrow), which was released at 18 atm after predilection by a 3.5 × 15 mm balloon. The proximal part of the stent was optimized with a 5 × 12 mm balloon.

We observed a 54-year-old man, a former chronic smoker, with no risk factors for sexually transmitted infections (STIs). His mother and wife have no history of STI, specifically syphilis and HIV. The patient has no risky behaviours either. No significant medical history and no previous episodes of recurrent angina in childhood, chancres, or skin rashes were found. He had been experiencing exertional dyspnoea without chest pain for 1 year, along with a decline in his overall health, which prompted him to seek emergency care due to acute respiratory distress. The cardiovascular examination revealed no abnormalities, except for a more pronounced diastolic murmur at the aortic focus. Peripheral pulses were present and symmetrical, and the neurological examination yielded normal results. An electrocardiogram showed sinus rhythm with lateral ST depression and *T*-wave inversion. Coronary angiography revealed a severe stenosis in the left main stem assessed by visual angiography, while the rest of the coronary network appeared completely smooth without any signs of atherosclerosis. The patient underwent angioplasty, resulting in a satisfactory outcome (*Figure 1A–D*), and subsequently received optimal treatment with dual antiplatelet therapy, statin, beta-blocker, renin-angiotensin-aldosterone system inhibitors, and sodium-glucose cotransporter-2 inhibitors.

**Figure 2:**
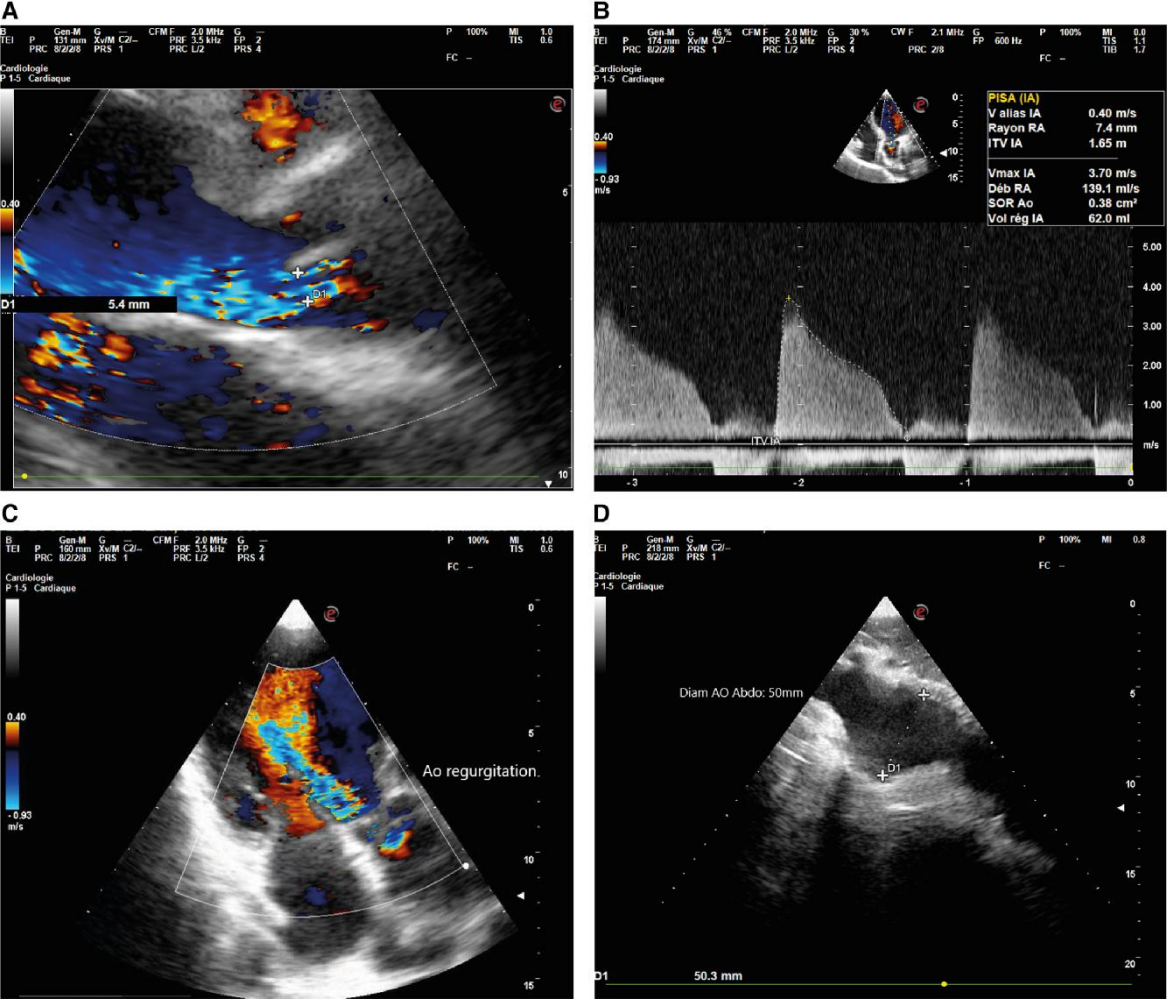
(*A*–*C*) Ultrasound image showing significant aortic insufficiency on a supple tricuspid valve, suggesting inflammatory damage to the valve. (*D*) Partially thrombosed dilation of the abdominal aorta.

During follow-up, the patient remained symptomatic, presenting with New York Heart Association (NYHA) Stage 3 dyspnoea but without angina or fever. An echocardiogram revealed a dysfunctional, globularly dilated left ventricle (LV) with an LV ejection fraction of 40%, a severe AI (aortic regurgitation area of 0.41 cm^2^; *Figure 2A–C*), a dilated ascending aorta measuring 41 mm, and a 50 mm abdominal aortic aneurysm (*Figure 2D*).

**Figure 3:**
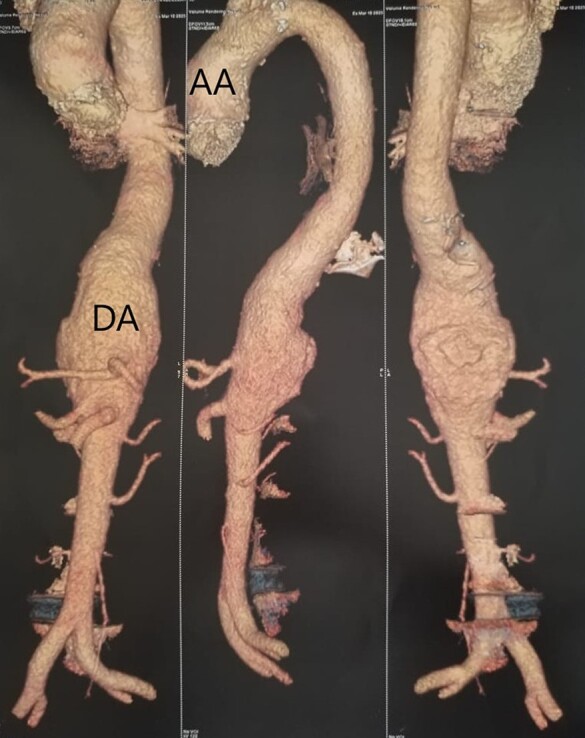
Aortic angiography images showing dilation of the suprarenal thoracic and abdominal aorta. AA, ascending aorta; DA, descending aorta.

Further evaluation using computed tomography angiography of the aorta disclosed fusiform dilation of the ascending thoracic aorta and cylindrical dilation of the suprarenal abdominal aorta, measuring 51 × 46 mm. This dilation was partially thrombosed over a 10 cm length with multiple parietal ulcerations (*Figure 3*).

In response to these findings, a biochemical assessment was conducted, revealing impaired renal function, estimated glomerular filtration rate at 40 mL/min/1.73 m^2^ (>90 mL/min/1.73 m^2^), an erythrocyte sedimentation rate four times the normal range for the first hour (<20 mm/h), negative C-reactive protein results (<8 mg/L), and positive syphilitic serology with treponema pallidum haemagglutination assay at a titre of 1/2560 and with venereal disease research laboratory test at a titre of 1/64, along with IgG and IgM antibodies measured at 39.

The syphilitic origin was confirmed through positive serological tests, consistent with a presentation of tertiary syphilis, and the absence of evidence supporting an alternative aetiology. Following a consultation with a cardiovascular surgeon and vascular team, it was decided to proceed with aortic valve replacement and aorto-coronary bypass surgery, with a mechanical aortic prosthesis On-X N 23 and double bypass of proximal interventriculaire antérieure and first marginal via the left internal mammary artery, but vascular surgery of the ascending aortic aneurysm was not possible at once, and conservative treatment with close monitoring was decided upon. The patient underwent the surgery within the week.

The patient was seen at the follow-up consultation 1 month after the operation. Residual NYHA Grade 2 dyspnoea was found. The local condition of the sternotomy scar was satisfactory, and no significant ultrasound gradient was found on the aortic valve. Two months after the operation, there was a complete improvement in the patient’s overall condition.

## Discussion

The syphilitic disease, when it produces cardiovascular symptoms and manifestations, is in its tertiary period, between 10 and 30 years after the primary infection in ∼30% of untreated patients. It affects the cardiovascular system in several ways. An aortitis is seen most frequently; this can lead to root dilatation, aneurysm formation, aortic regurgitation, and stenosis of the coronary ostia. Several of these clinical manifestations may coincide, but when associated with the presence of an aneurysm, the mortality, if not repaired surgically, reaches 80% at 2 years.^3^ Pathologically, there is an endarteritis of the vasa vasorum associated with a mesoaortitis, a patchy loss of elastic tissue, and a fibrous replacement. Exceptionally, rare manifestations of cardiovascular syphilis include myocardial gumma and coronary arteritis. Cardiac involvement is more common in men, and there is also a reported association with systemic hypertension.^4^

The diagnosis will be evoked in the presence of certain clinical signs and in a particular epidemiological context; it is routinely based on serological methods. Direct methods remain the prerogative of specialized structures.

Syphilitic aortitis is the most common cardiovascular disease in tertiary syphilis. It is due to a migration of the spirochaetes towards the media through the lymphatics of the vascular wall shortly after the primary infection. These lymphatics are more frequent at the level of the ascending and horizontal aorta, which explains the preferential localization of aortic aneurysms at their levels. Vasculitis of the vasa vasorum ensues, resulting in patchy necrosis of the media with the destruction of the elastic fibres and installation of fibrosis. The aortic wall is thus weakened, subjected to the high-pressure regime, which prevails in the aorta, and will gradually dilate with the formation of most often sacciform aneurysms.^5^

The diagnosis of syphilitic aneurysm is made in the presence of an ‘atypical' aneurysm unrelated to an atheromatous cause, localized at the level of thoracic aorta, rarely abdominal, and sacciform, in patients without a history of arterial hypertension and presenting serology of syphilis active.^2^

Surgical intervention remains the gold standard for treating aortic aneurysms, particularly when the aneurysm exceeds a certain size or exhibits rapid growth. Open surgical repair, endovascular stent grafting, or a hybrid approach may be considered based on individual patient factors and anatomical considerations. The simultaneous presence of aortic regurgitation or significant coronary disease should be surgically treated at the same time.^6^

Coronary ostial stenosis is seen in 0.13–2.7% of coronary attacks and represents the second cardiovascular complication of syphilis.^7^

The coronary involvement in our patient was exclusively left coronary orifice and contrasts with a perfectly healthy underlying coronary network; these ostial involvements are frequently observed in wearers of aortic prostheses^8^ or people with inflammatory vasculitis such as Takayasu^9^ or syphilis.^10^ It is this particularity, as well as the results of the serology, that allowed us to establish the diagnosis of syphilitic coronary even in the absence of histological proof.

It is important to emphasize the aetiology of inflammatory diseases including syphilis in the differential diagnoses of patients with coronary ostial lesion and normal distal bed. Primary percutaneous coronary intervention with stenting can be a safe and effective alternative to coronary artery bypass graft in the case of these non-atherosclerotic lesions.^11^

The place of percutaneous angioplasty in this pathology lacks data and requires more studies. Some authors report their experiences of syphilitic ostial coronary treated by percutaneous angioplasty with modest results, especially the recurrence of anginal attack and stent stenosis after a short period.^12^

The syphilitic origin of an aortic aneurysm may be suggested by the sacciform shape, frequently surrounded by an inﬂammatory periarterial wall, and the topography of the lesion, often at the ascending aorta and the aortic arch; this link remains difficult to establish when it is a question of an AI, which raises the differential diagnosis mainly with infective endocarditis and must be eliminated before retaining the diagnosis of syphilitic AI in particular by clinical, haematological, and/or imaging arguments. However, it is possible that the two pathologies coexist.^13^

Even with significant clinical improvement in the patient with AI under medical treatment, prosthetic valve replacement surgery should be considered as recommended by Otani *et al*.^14^

The coexistence of an ostial left coronary stenosis and an AI of syphilitic origin imposes an initial treatment with penicilin G followed by aortic valve replacement surgery associated with a coronary bypass as was the experience of Otani *et al*.^14^

The various cardiovascular complications of syphilis can coexist in the same patient.^15^ As in our observation, the patient suffers from all three diseases: syphilitic aortitis, ostial coronary, and AI.

## Conclusion

Our findings underscore the importance of considering this exceptional pathology in cases of ostial coronary in a young person, a mycotic aneurysm of the aorta, and/or long-standing AI after ruling out the most common differential diagnoses, especially infective endocarditis, which may coexist in rare instances.

## Lead author biography


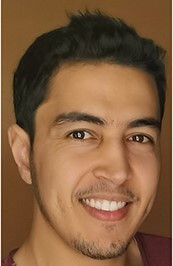


Dr Taha Berhil: born on 27 April 1993, of Moroccan nationality, married, and a father of one child. In 2019, he received his doctor’s degree in general medicine from the Faculty of Medicine of Monastir Tunisia and accreditation to practice family medicine. In 2021, he was an intern in cardiology at the University Hospital Mohammed VI, Tangier, Morocco.

## Supplementary Material

ytae013_Supplementary_Data

## Data Availability

The data underlying this article are available in the article and in its Supplementary material online.
